# Decoding covert shifts of attention induced by ambiguous visuospatial cues

**DOI:** 10.3389/fnhum.2015.00358

**Published:** 2015-06-18

**Authors:** Romain E. Trachel, Maureen Clerc, Thomas G. Brochier

**Affiliations:** ^1^CNRS, Institut de Neurosciences de la Timone, Aix-Marseille UniversityMarseille, France; ^2^INRIA Sophia Antipolis-Méditerranée – Athena Project TeamSophia Antipolis, France; ^3^Laboratoire des Sciences Cognitives et Psycholinguistique, Ecole Normale SupérieureParis, France

**Keywords:** visuospatial attention, cues, decoding, electroencephalography, brain–computer interface, spatial filtering

## Abstract

Simple and unambiguous visual cues (e.g., an arrow) can be used to trigger covert shifts of visual attention away from the center of gaze. The processing of visual stimuli is enhanced at the attended location. Covert shifts of attention modulate the power of cerebral oscillations in the alpha band over parietal and occipital regions. These modulations are sufficiently robust to be decoded on a single trial basis from electroencephalography (EEG) signals. It is often assumed that covert attention shifts are under voluntary control, and that they also occur in more natural and complex environments, but there is no direct evidence to support this assumption. We address this important issue by using random-dot stimuli to cue one of two opposite locations, where a visual target is presented. We contrast two conditions, one in which the random-dot motion is predictive of the target location, and the other, in which it provides ambiguous information. Behavioral results show attention shifts in anticipation of the visual target, in both conditions. In addition, using the common spatial patterns (CSPs) algorithm, we extract EEG power features in the alpha-band (around 10 Hz) that best discriminate the attended location in single trials. We obtain a significant decoding accuracy in 7/10 subjects using a cross-validation procedure applied in the predictive condition. Interestingly, similar accuracy (significant in 5/10 subjects) is obtained when the CSPs trained in the predictive condition are tested in the ambiguous condition. In agreement with this result, we find that the CSPs show very similar topographies in both conditions. These results shed a new light on the behavioral and EEG correlates of visuospatial attention in complex visual environments. This study demonstrates that alpha-power features could be used in brain–computer interfaces to decode covert attention shifts in an environment containing ambiguous spatial information.

## Introduction

In anticipation of an upcoming target in the visual periphery, the focus of attention can be voluntarily and covertly shifted away from the gaze direction (Corbetta and Shulman, 2002). Covert visuospatial attention has been extensively studied in experimental paradigms in which a spatial cue (e.g., symbol, arrow, letter) presented at a central fixation point, indicates the peripheral location of an upcoming target (Posner, 1980; Dosher and Lu, 2000; Jongen et al., 2006; Thut et al., 2006; Green and McDonald, 2008, 2010; Wyart and Tallon-Baudry, 2008; Yamagishi et al., 2008). In anticipation of the target presentation, subjects voluntarily shift their attention according to the cue, leading to shorter reaction times and more accurate responses to targets presented at attended (cued) locations than at non-attended ones (uncued). Moreover, electroencephalography (EEG) and magnetoencephalography (MEG) recordings suggest that the covert shifts of attention modulate oscillatory activity over the parietal and occipital lobes. Alpha power (e.g., around 10 Hz) is suppressed over the hemisphere controlateral to the attended visual field (Yamagishi et al., 2003, 2005; Rihs et al., 2007; Wyart and Tallon-Baudry, 2008; Green and McDonald, 2010) and increased over the ipsilateral hemisphere (Worden et al., 2000; Sauseng et al., 2005; Thut et al., 2006; Rihs et al., 2009; Cosmelli et al., 2011). Importantly, these attention-related modulations of alpha power are sufficiently robust to be decoded on a single trial basis (van Gerven and Jensen, 2009; Bahramisharif et al., 2010; Treder et al., 2011) with up to 90% accuracy for protocols in which targets are presented to a left vs. right location (Treder et al., 2011; Roijendijk et al., 2013; Tonin et al., 2013).

It has been shown that in the absence of spatial cue, visuospatial attention can be covertly shifted under volitional control (Hopfinger et al., 2010) and alpha power modulations can be used to decode the attended location (Bengson et al., 2014). These observations lead to the assumption that covert attention shifts could also be decoded in complex environments, for instance in a driving situation while gazing at the road and shifting attention to the rear-view mirror. However, unlike experimental approaches in which unambiguous cues are used to induce attention shifts, such real-life situations rarely contain information pointing directly toward specific target locations. Alternatively, they provide multiple cues that are used by the subject to shift attention in a direction that may not be predictable from the visual scene. This striking difference between experimental and real-life situation raises two main issues.

First, it has not been demonstrated so far that subjects can produce voluntary shifts of visual attention when the visual scene is complex and contains ambiguous directional cues. Second, it is not known how brain correlates of attention shifts in a complex visual environment compare to those induced by unambiguous cues and if they can be decoded in the same way.

In order to approach these issues, it is critical to evaluate how visuospatial attention is shifted following the presentation of complex visual cues. In the present study, we designed a task in which the subject must extract directional information from a random dot motion stimulus, and then shift attention in the indicated direction, toward a location where a visual target is going to appear, and will have to be discriminated. The directional information to be extracted from the random dot stimulus is either right or left, corresponding to the two possible target locations. We investigated two experimental conditions. In the predictive condition, the direction of dot motion is biased toward the left or the right, and the subject can reliably predict the location of the target. In the ambiguous condition, the direction of dot motion is uniformly distributed and the subject is unable to predict where the visual target will appear. Importantly, the two conditions are randomly alternated and the subject is not informed that some of the cues are ambiguous.

We hypothesize that in the ambiguous condition, the subject will tend to use isolated local information in the complex cue, in order to select where to shift attention. We also hypothesize that in this condition, in which the direction of attention shift is unpredictable from the visual cue, neural signals represent the only reliable source of information to decode the location of covert attention. This paper analyzes both behavioral performance and EEG correlates of visuospatial attention. We confirm that attention shifts produced in the ambiguous condition are comparable to those produced in the predictive condition. Finally, we discuss the potential of using such modulations for the development of covert visuospatial attention brain–computer interface (BCI). This paper makes a seminal contribution to the literature of visuospatial attention by showing that EEG signals can be used to decode the location of covert attention shifts in situations where the environment contains ambiguous information.

## Materials and Methods

### Participants and Apparatus

Ten male subjects aged between 21 and 44 (mean 26.9) with normal or corrected-to-normal vision participated in the experiment. All subjects provided informed written consent and were paid for their participation in the study, which was approved by the local ethics committee of Aix-Marseille University. EEG was recorded from a BioSemi ActiveTwo system, from 64 electrodes mounted on an elastic cap according to the 10–20 method (1024 Hz sampling rate). In addition, eye movements were recorded by electrooculography (EOG) with bipolar montage, measuring voltage differences between two external electrodes fixed at the outer canthi of each eye (horizontal component), and between the Fp2 electrode and another external electrode fixed below the right eye (vertical component). The task was designed in Matlab using the PsychToolbox package (Brainard, 1997) for visual display and the Data Acquisition toolbox for recording behavioral responses from a custom-made two-button box connected to a National Instrument data acquisition card. Data were analyzed on a high performance computing cluster using MNE (Gramfort et al., 2014) and scikit-learn (Pedregosa et al., 2011) for EEG processing and feature classification.

### Procedure

**FIGURE 1:**
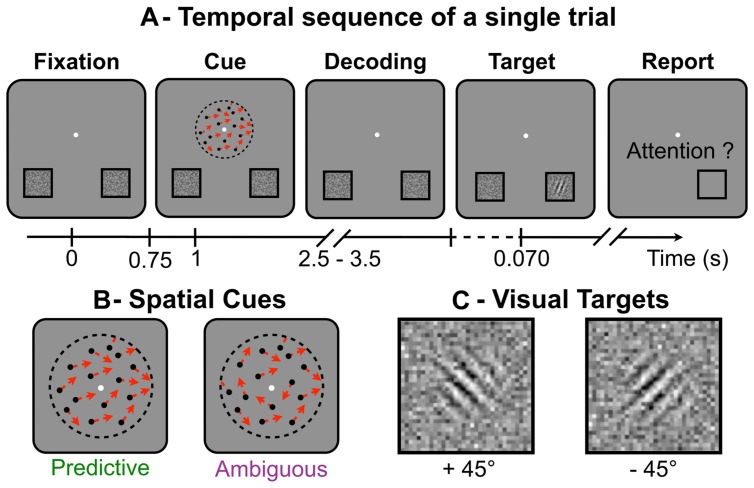
**(A)**Temporal sequence of a single trial: while fixating the central white dot (A-Fixation), subjects had to distinguish the global direction of the random-dot motion (A-Cue), to shift their attention toward the location corresponding to the global direction (A-Decoding), to react to the target to identify its orientation (A-Target) and, last, to report if they had indeed attended the location where the target appeared (A-Report). **(B)** Schematic representation of the spatial cues made of random-dots (single directions shown as red arrows), which appear for 250 ms. **(C)** The visual targets (Gabor tilted at ±45° and embedded in noise), here displayed with higher contrast than in the actual experiment. Targets were briefly flashed (70 ms) and the subjects had to report on their orientation.

During the experiment, the subjects performed a visuospatial attention task in a modified version of Posner’s paradigm (Posner, 1980; Dosher and Lu, 2000). For this task, the subjects had to keep fixating a central point throughout the trials. All trials followed the same temporal sequence (**Figure 1A**). First, the subjects had to use a random-dot spatial cue (**Figure 1B**) presented at the fixation point to decide on the location where to anticipate a forthcoming target (displayed at the lower-left or lower-right part of the screen). Second, they were instructed to shift their attention toward the cued location, where the visual target (**Figure 1C**) was briefly flashed (70 ms) after a random delay of 1.5–2.5 s following the cue presentation. Finally, they had to detect and identify as fast and as accurately as possible the target orientation with respect to the vertical axis. The subjects responded by a right- or left-thumb button press to indicate whether the target was tilted at plus or minus 45° respectively. They had to respond within 3 s after target presentation. Button responses were used to compute reaction time (RT) and error rate (ER) for target identification. The task was designed so that the direction indicated by the random-dot motion was difficult to discriminate and in some trials, the subjects shifted attention to the side opposite to target presentation, making a so-called “spatial error.” At the end of each trial, the subjects reported with the button box if the target had appeared at the location they had actually attended. Using this subjective report, we computed the spatial ER for motion discrimination. The target locations and orientations were pseudo-randomly balanced in order to minimize sequential and priming effects on RT and ER. Overall, the session included a training period (52 trials), two adaptive procedures to set the cue and target parameters (40 trials each) and the recording session (eight blocks of 52 trials, interleaved with short breaks).

### Visual Stimuli

The spatial cues and the visual targets were displayed on a 50 cm CRT monitor (resolution 800 × 600, 140 Hz) 60 cm away from the subject’s eyes. The spatial cue consisted of 400 dots, each moving in a single direction for 250 ms, within a 5° circle around the central fixation point. In each trial, the dot directions were randomly drawn from a Von Mises distribution whose mean indicated the target location and whose variance was the inverse of motion coherence. We used the QUEST procedure (Watson and Pelli, 1983) to measure the threshold coherence value allowing each subject to achieve the task with a spatial ER of 10%. During this procedure, random-dot motion coherence was adapted from trial to trial in a stimulus-response sequence that enabled fast computation of the subject’s coherence threshold after 40 or more trials, according to convergence criteria. This adaptive procedure ensured that all subjects had a similar level of performance in motion discrimination.

Two experimental conditions were used. In the predictive condition, we set the coherence at threshold so that subjects could reliably extract the average direction contained in the dots motion in about 90% of the trials (10% spatial ER). In the ambiguous condition, the motion coherence was set to zero. The resultant direction of the dots motion had a vector norm <0.05 and was expected to be perceived at chance level by the subjects (spatial ER around 50%). In both conditions, the target location was determined by the average direction of the dots.

During the task, the subjects underwent overall 75% of predictive and 25% of ambiguous trials, pseudo-randomly interleaved. In combination with the spatial ER expected from the adaptive procedure, these proportions were such that the target was eventually displayed at the non-attended locations in about 20% of the trials (75^∗^0.1+25^∗^0.5). The overall 20% spatial ER was critical to the goal of the experiment (Posner, 1980). This low value ensured that all subjects adopted a strategy in which they voluntarily shifted attention to one of the two possible target locations, rather than dividing attention between them (Cosmelli et al., 2011). Importantly, to ensure that they would use a similar strategy in both the predictive and ambiguous conditions, they were not informed that ambiguous trials would occur in the experiment. At the end of the protocol, the subjects where questioned about the experiment. None of them noticed that two different types of cue were used in the experiment but they noticed that sometimes, the direction of the dots motion was difficult to perceive. Therefore, we assume they were voluntarily shifting attention to the cued location, or randomly choosing one location when they felt unsure about the main direction of the random-dot motion.

The visual target consisted of a Gabor pattern tilted at ±45° and presented for 70 ms at the center of one of two squares located at ±9.23° horizontal and -5° vertical eccentricity from the central point (Bahramisharif et al., 2011). A dynamic noise was displayed inside both squares for the entire duration of the trial (Dosher and Lu, 2000). The noise contrast was displayed at 35% of maximum intensity with a spatial resolution 10 times lower than the target. For each subject, Gabor contrast was set below the noise contrast using another QUEST procedure (Watson and Pelli, 1983) to ensure that the ER would be around 10% when targets were displayed at the attended location, and around chance level (50%) at the unattended location. During this adaptive procedure, the target location was cued by an arrow instead of a random-dot motion to ensure the subjects would shift their attention to target location in every trial. Moreover, the target locations (lower left and right) and orientations (±45°) were pseudo-randomly balanced in order to minimize sequential and priming effects.

### EEG Data Analysis

Raw EEG signals were re-referenced to the common average reference and filtered in the alpha range (8–14 Hz) with a forward–backward band pass filter (Butterworth, order 4). Single trials were epoched into time windows of 1.5 s before target onset. Epochs were rejected when the EOG signal reached a threshold of 100 μV for the vertical and 40 μV for the horizontal component (Rihs et al., 2009). After EOG rejection, on average 307.6 ± 3.6 predictive and 102.8 ± 1.2 ambiguous trials per subject were available for further analysis. The subject’s report was used to label each epoch in two classes (left and right shifts of attention). This pre-processing step resulted in a set of EEG signals X_eeg_ ∈ R^N×T^ with *N* = 64 electrodes and T = 1536 (1024 × 1.5) time samples for each epoch. Additionally, a set of EOG signals X_eog_ ∈ R^2×T^ was analyzed in order to subsequently verify through decoding that behavioral features (eye movements) could not be used to predict the direction of attention shifts.

A regularized version of the common spatial pattern (CSP) algorithm (Lotte and Guan, 2011) was applied to extract discriminant alpha-band power features of left vs. right shifts. The CSP algorithm is a spatial filtering method commonly used in motor imagery BCI for robust single-trial classification of EEG oscillatory activity (Blankertz et al., 2008). It has been shown recently that this method improves decoding performance in visuospatial attention BCI-based protocols (Fujisawa et al., 2008). The CSP algorithm is a data-driven projection method that maximizes the variance of spatially filtered signals X_csp_ ∈ R^N×T^ for one class of trials while minimizing it for the other. It computes a set of spatial filters W = {w_j_ ∈ R^N^}_j__=__1,...,N_ by solving the generalized eigenvalue problem:

$$\Sigma_{r}W=\lambda \Sigma_{1}W$$

with Σ_r_ and Σ_l_, the averaged covariance of the EEG signals X_eeg_ across right and left trials, respectively. Following (Lotte and Guan, 2011), we estimated the covariance matrix of the signals with an algorithm that computes the L2-norm regularization parameter using an optimal shrinkage approximation (see Pedregosa et al., 2011). Then, the EEG signals were projected from the original sensor space onto a surrogate space by X_csp_ = W^T^X. Finally, the CSP features were computed as the log variance of these spatially filtered signals for each single-trial epoch by log10(var(X_csp_)). Interestingly, the CSP algorithm provides neuro-physiologically interpretable information (Blankertz et al., 2008) for each feature, through the matrix A = (W^T^)^-1^ which characterizes the spatial patterns of the sources passing through each filter. In addition, EOG features were computed as the average signal value for each epoch. Both CSP and EOG features were independently standardized by mean subtraction and scaling to unit variance independently.

The single-trial epochs were decoded by a regularized linear support vector machine (SVM). Features were selected on the predictive trials using a recursive feature elimination procedure (Guyon et al., 2002) which has previously been applied in BCI for EEG channel selection (Lal et al., 2004). Basically, the recursive feature elimination procedure recursively prunes and ranks features given the weights assigned to them by SVM from a set of trials, then tested on a separate set of trials to compute the SVM cost function (i.e., hinge loss). This procedure was repeated in parallel over a 10-fold cross validation for a range of regularization parameters C = {c_i_ ∈ log10 space from -5 to 5}_i__=__1,…,100._ For each cross-validation iteration, the recursive feature elimination was applied to a set of training trials for 9/10 of the folds and tested on trials from the test set for the remaining fold. Finally, the optimal feature set and regularization parameter were selected to minimize the cost function averaged over the 10 test folds. The SVM was trained on the predictive trials with the selected feature set and regularization parameter, and tested to decode the ambiguous trials. The decoding performance was evaluated by computing the classification accuracy (rate of correctly classified trials) obtained across 10 test folds of predictive trials. Moreover, we also investigated the decoding performance in predictive features using an inner 10-fold cross-validation. For each train fold of predictive trials, we computed the CSP filters, applied the recursive feature elimination/cross-validation procedure and trained the SVM with the selected features and parameter. Importantly, features were always centered and scaled with the mean and the variance computed on the training set. The statistical significance of classification accuracy was evaluated from a binomial cumulative distribution (Waldert et al., 2008). The probability *p(k/n)* of classifying the correct location in at least *k* out of *n* trials by chance was computed. Classification accuracy *k/n* was considered significant if *p(k/n)* was lower than 0.05.

## Results

### Behavioral Performance

On average across subjects, target contrast and random-dot motion coherence thresholds were equal to 17.80% (±7.23 SD) and 1.29 (±0.26) respectively. As expected, target contrast was adapted below the noise contrast [1 sample *t*-test, mean at 35%, *t*(9) = -7.131; *p* = 5.475e-5]. In this condition, we assumed that a lateral shift of attention was the optimal strategy to reduce the noise effect (Dosher and Lu, 2000) and to produce fast and accurate behavioral responses to the target.

#### Spatial Errors

**FIGURE 2:**
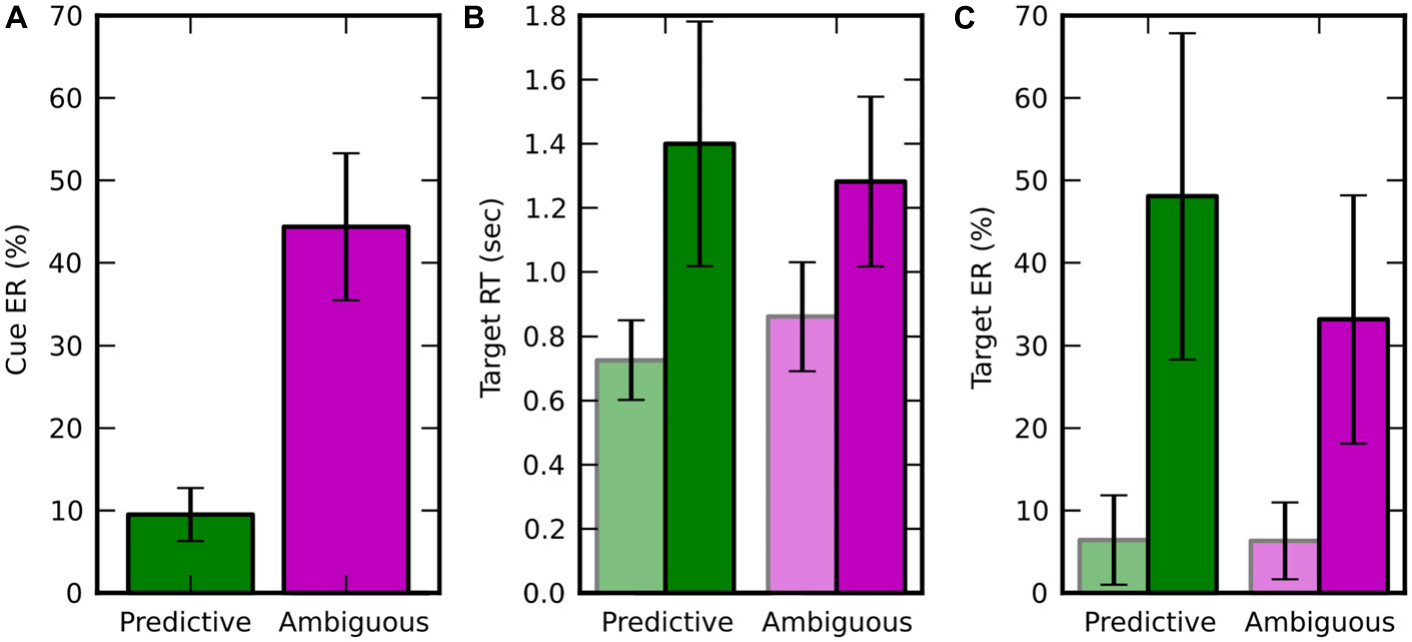
**Behavioral results, displayed in green for predictive cues and in pink for ambiguous cues.** Trials in which the target appeared at attended (resp. unattended) location are in light (resp. dark) colors. Error bars represent the SD over 10 subjects. **(A)** Averaged spatial error rate (Cue ER), i.e., error when estimating the target location from the random moving dots. **(B)** Average target response time (Target RT; **C**) Average target ER (Target ER).

The average spatial ER across subjects in discrimination of random-dot motion direction (left vs. right) was 18.23 ± 2.78%, i.e., very close to the 20% rate expected from the coherence thresholds adaptation (see Materials and Methods). In ambiguous trials, the spatial ER was not significantly different from chance level [50%, 1 sample *t*-test, *t*(9) = -1.894; *p* = 0.091]. This result confirms that the average direction of the dot motion in the ambiguous condition was so low (resultant norm < 0.05) that it was not reliably perceived by the subject and wasn’t predictive of the direction of attention shifts. The spatial ER was significantly lower for predictive than ambiguous cues [**Figure 2A**, paired *t*-test, *t*(9) = -11.236; *p* = 5.408e-7]. All these effects were in agreement with to the performance expected from the adaptive procedure that set random-dot motion coherence thresholds for each subject to produce 10% spatial ER with predictive cues.

#### RT and Target Errors

In the target discrimination task, when the target appeared both RT and ER were significantly lower at the attended location rather than at the unattended one (**Figures 2B,C**). For RT, we found a main effect of visuospatial attention [*f* (1,9) = 57.280, *p* = 3.438e-05] and an interaction with the cueing condition [*f*(1,9) = 15.367, *p* = 0.0035] using a two-way repeated measure ANOVA (factors: attended/unattended and predictive/ambiguous). For ER, we found a main effect of visuospatial attention [*f*(1,9) = 47.574, *p* = 7.088e-05] and also a main effect of cueing condition [*f*(1,9) = 11.478, *p* = 0.008], but no interaction between these two factors [*f*(1,9) = 4.478, *p* = 0.063]. In addition, the behavioral performance for trials, pooled independently of the attention location, was lower for predictive than for ambiguous cues for both RT [0.789 ± 0.146 s vs. 1.047 ± 0.203 s, *t*(9) = -5.238; *p* = 0.000536] and ER [10.081 ± 5.241% vs. 33.147 ± 15.031%, *t*(9) = -3.616; *p* = 0.0056].

#### Performances of Individual Subjects

We questioned whether the behavioral performance of individual subjects was related to their random-dot motion coherence threshold or their target contrast threshold, as specified in the adaptive procedures. We first investigated the correlation [Spearman’s rank (*r*)] between coherence thresholds and spatial ERs, and found no significant correlation across subjects. We then computed the correlation between target contrast and the subjects’ performance in response to the visual target (RT and ER). We did not find any correlation between target contrast and RT (*n* = 10, *r* = -0.139; *p* = 0.701 for predictive nor *r* = 0.357; *p* = 0.310 for ambiguous trials), but we did find a correlation between target contrast and ER both for predictive (*n* = 10, *r* = -0.688; *p* = 0.0279) and for ambiguous trials (*n* = 10, *r* = -0.673; *p* = 0.0330). These results reveal that the target ER was determined in part by the subject’s performance in the adaptative procedure for target contrast.

### Spatial Patterns

**FIGURE 3:**
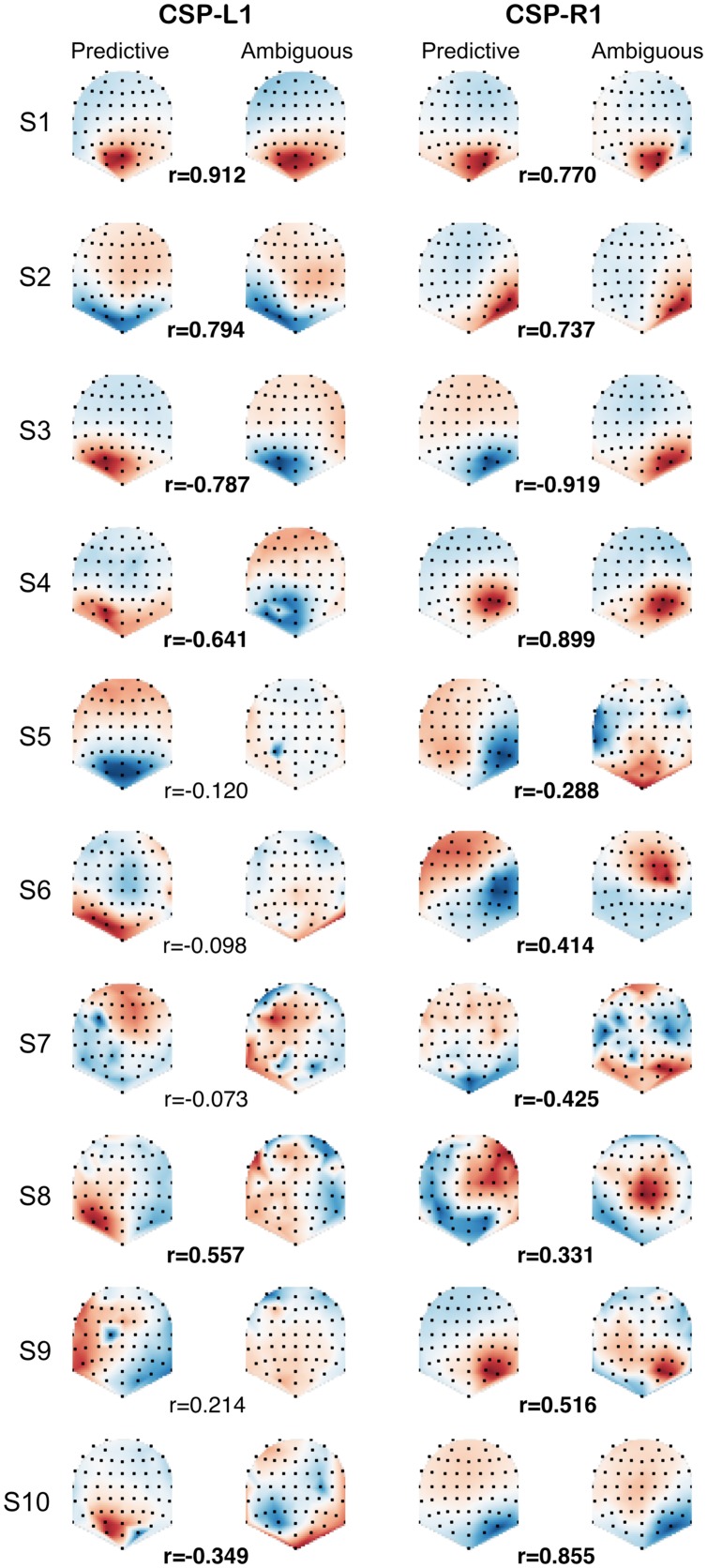
**CSP-L1 vs. CSP-R1 are spatial patterns corresponding to the most discriminant spatial filters for trials in which attention is shifted to the left vs. right location, respectively.** The CSPs are fitted in either predictive (first and third column) or ambiguous trials (second and fourth column) for each subjects (rows). The pattern coefficients are topographically mapped onto subjects’ scalp from blue to red (sign and scale are irrelevant). Correlation coefficients (*r*) between predictive and ambiguous spatial patterns are indicated for each subject, with significant *r* values in bold.

In order to compare the spatial patterns of brain activity induced by predictive and ambiguous cues, we fitted the CSP filters separately on predictive and ambiguous epochs for each subject. We investigated the relationship between predictive and ambiguous spatial patterns A = {a_j_ ∈ R^N^}_j__=__1,...,N_ for which the coefficients (a_j_) are topographically mapped onto the subject’s scalp. We defined the spatial patterns corresponding to the most discriminative filters of each class as CSP-L1 and CSP-R1, respectively. These patterns are shown in **Figure 3** for each subject and each condition. Note that the signs of the pattern coefficients can be flipped between subjects, because for CSP filters, sign is arbitrary (Blankertz et al., 2008). The largest coefficients in absolute value were distributed either over the left or the right posterior sensors. Interestingly, we observed very similar patterns when comparing filters between predictive and ambiguous conditions for the majority of the subjects (**Figure 3**). To assess the significance of these observations, we computed a within-subject Spearman’s correlation (*r*) between the coefficients of predictive and ambiguous spatial patterns. The results showed significant *r* values (*n* = 64, *p* < 0.05) in six subjects (S1, S2, S3, S4, S8, and S10; mean *r*^2^ = 0.616) for CSP-L1 and in all subjects (mean *r*^2^ = 0.615) for CSP-R1. These correlations between the spatial patterns fitted in single-trial predictive and ambiguous epochs suggest that alpha-band EEG oscillations that best discriminate the attended location shared common topographies in both conditions. Next, we compared the information contained in each feature set with respect to the spatial location of attention.

### Single-Trial Power Modulations

**FIGURE 4:**
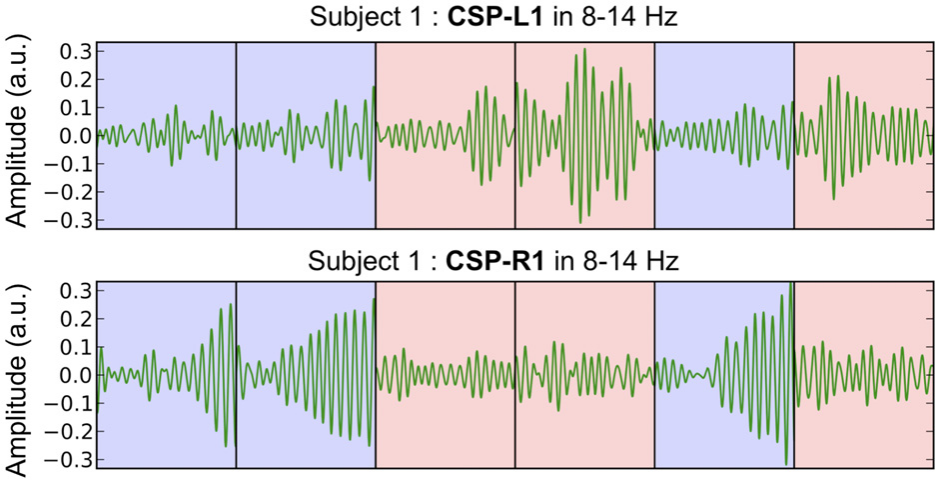
**Concatenated epochs of ambiguous trials from Subject 1 in which the variance of the spatially filtered signals (CSP-L1 and CSP-R1, fitted on predictive trials) is maximized for one category of trials and minimized for another category of trials.** The light blue and red background corresponds to right and left shifts of attention, respectively.

The most discriminant filters produced clear power modulations on a single-trial basis with respect to the shift of attention (left vs. right). **Figure 4** shows the concatenation of single-trial ambiguous epochs from subject 1, filtered with CSP-L1 (top) and CSP-R1 (bottom). The power of alpha-band oscillations was higher at the output of CSP-L1 than CSP-R1 when attention was shifted to the left (**Figure 4**, red epochs). The opposite modulations could be observed when attention was shifted to the right (**Figure 4**, blue epochs). Indeed, the spatial patterns of the filters suggested a dominance of the posterior sensors ipsilateral to the attention shifts. We tested the significance of these observations for each subject with a Student *t*-test computed between standardized features of the right vs. left single-trial ambiguous epochs. Importantly, the first and last filters used to extract these features were fitted only on predictive epochs (see Materials and Methods). The results showed significant *t*-values (*p* < 0.05) in four subjects (S1, S2, S3, S4, and S5; mean *t* = -3.391) for CSP-L1 and in six subjects (S1, S3, S4, S6, S9, and S10; mean *t* = 4.102) for CSP-R1.

### Decoding Performance

**FIGURE 5:**
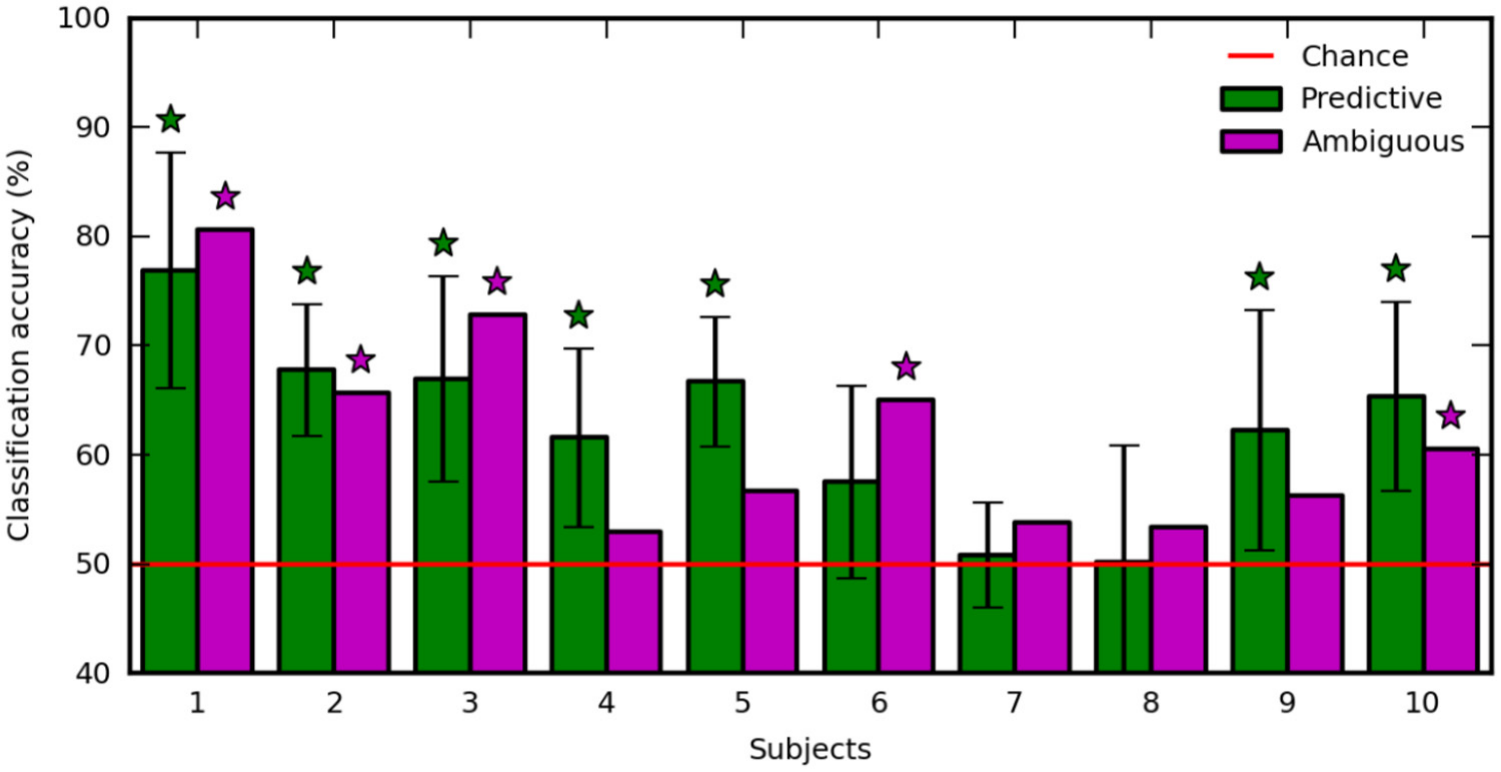
**Classification accuracy with predictive (green) and ambiguous (purple) features for each subject.** The red line indicates chance level. Error bars are the SD over 10 runs of cross-validation. Stars indicate statistically significant results (*p* < 0.05).

The regularization parameter and the feature set used to decode ambiguous trials were selected in a 10-fold recursive feature elimination/cross-validation procedure applied in predictive trials. Also, we decoded predictive trials using an inner 10-fold recursive feature elimination/cross-validation procedure (see Materials and Methods). **Figure 5** shows the classification accuracy computed in ambiguous trials (**Figure 5**, purple), and averaged over the 10-fold cross-validation in predictive trials (**Figure 5**, green). The decoding performance was similar in predictive and ambiguous trials [62.60 ± 8.12% vs. 61.79 ± 9.24%, paired *t*-test, *t*(9) = 0.412; *p* = 0.690] with up to 76.90 and 80.58% for S1, respectively. It is important to note that, even with the ambiguous cues, the average accuracy was comparable with the performance reported in earlier studies of covert-attention BCI, which systematically used predictive cues (van Gerven and Jensen, 2009; Bahramisharif et al., 2010; Treder et al., 2011; Roijendijk et al., 2013; Tonin et al., 2013). The classification accuracy was significant for seven subjects with predictive cues (S1, S2, S3, S4, S5, S9, and S10) and for five subjects with ambiguous cues (S1, S2, S3, S6, and S10). Indeed, we found a positive correlation in the classification accuracy of predictive and ambiguous features (*n* = 10, *r* = 0.794; *p* = 6.10e-3). However, three subjects (S3, S4, and S5) still showed a significant difference in classification accuracy for decoding predictive and ambiguous features [1 sample *t*-test, *t*(9) = 3.162; *p* = 0.0115 for S3, *t*(9) = 5.017; *p* = 7.217e-04 for S4, and *t*(9) = -2.544; *p* = 0.0315 for S5]. As recently shown in the literature of covert attention based BCI (Roijendijk et al., 2013), target contrast and classification accuracy are negatively correlated across subjects, both for predictive (*n* = 10, *r* = -0.600; *p* = 0.067) and for ambiguous (*n* = 10, *r* = -0.224; *p* = 0.533) cues. This result suggests that at lower contrast, the subjects must shift attention more purposefully to detect the target (Dosher and Lu, 2000). In order to ensure that eye movements were not predictive of left vs. right attention shifts, we also applied our decoding analysis to EOG features extracted in predictive and ambiguous trials. Classification accuracy was near chance level for predictive (52.01 ± 2.69%) and ambiguous (49.87 ± 4.93%) trials, confirming our hypothesis that, in ambiguous cueing conditions, neural signals represent the only reliable source of information to decode the location of covert attention.

## Discussion

There is experimental evidence showing that covert attention shifts are under volitional control after presentation of an unambiguous cue (Worden et al., 2000; Hopfinger et al., 2010), even when the cue contains no spatial information (Cosmelli et al., 2011; Bengson et al., 2014). In the present paper, we demonstrated that subjects can shift attention to specific locations following the presentation of random-dot motion cues containing ambiguous spatial information. This result reveals that conflicting spatial cues contained in the ambiguous random-dot pattern do not interfere with the subject’s ability to attend a specific spatial location, when asked to do so. In addition, our EEG recordings revealed striking similarities between the spatial patterns of brain activity extracted in the alpha band from covert shifts of attention induced either by predictive or ambiguous random-dot motion cues. Following both types of cues, the EEG signals could be decoded with up to 80% accuracy to discriminate between left and right attention shifts. Our results suggest that human subjects were able to voluntarily and covertly shift attention in a well-controlled experimental context containing ambiguous spatial information. This point is crucial for the future development of BCIs by providing evidence that covert attention can voluntarily be shifted in complex visual environments.

### Cueing Effects on Behavioral Performance

The behavioral results demonstrate that subjects shifted attention location even if they could not reliably discriminate the global direction motion of the cue. When the subjects reported the target to appear at the attended location, the behavioral response was faster and more accurate than when it had appeared at the unattended location (Posner et al., 1980). Interestingly, this effect was observed both for predictive and ambiguous cueing conditions and with a similar strength (**Figure 2**). This suggests that for both types of cues, the processing of low contrast target embedded in noise (Dosher and Lu, 2000) is enhanced with covert shifts of attention. Previous studies have already demonstrated that unambiguous spatial cues (i.e., an arrow) triggers cueing effects, whether the cue is weakly (Dosher and Lu, 2000; Worden et al., 2000; Yamagishi et al., 2003, 2005; Thut et al., 2006; Wyart and Tallon-Baudry, 2008) or highly informative (>75%; Posner, 1980; Posner et al., 1980; Sauseng et al., 2005; Rihs et al., 2007, 2009; Cosmelli et al., 2011) about the target location. Here, we showed alternatively that if the spatial cue is highly informative about the target location, i.e., when the average spatial ER is maintained around 20% across all predictive and ambiguous trials (Posner, 1980), subjects shifted attention even in totally ambiguous conditions containing unreliable spatial information about the target location. One likely interpretation is that in the absence of obvious directional information in the ambiguous condition, subjects relied on local motion information to select one of the two target locations. This strategy was strengthened by the fact that the subjects were not told about the ambiguous cues, and they were not able to not distinguish between the two types of cues. In their subjective reports at the end of experiment, subjects noted that the direction of the random-dot motion was often difficult to discriminate but they didn’t report about different cueing conditions (i.e., predictive vs. ambiguous).

Two questions could be investigated in future studies. First, it would be important to know if attention shifts in ambiguous trials still occur when the proportion of ambiguous trials is increased. As in earlier works (Dosher and Lu, 2000; Worden et al., 2000; Yamagishi et al., 2003, 2005; Thut et al., 2006; Wyart and Tallon-Baudry, 2008), this approach would increase the average spatial ER and decrease the information content of the cue. Second, we would need to test if similar attention shifts occur when the ambiguous cue is replaced by a cue containing no spatial information at all, like a circle appearing at the fixation point. However, we think that the visual presentation of a neutral cue would lead to temporal rather than spatial attention shifts (Jongen et al., 2006; Cosmelli et al., 2011).

### Spatial Patterns of Brain Activity

The spatial filters used for feature extraction and classification in BCI (Blankertz et al., 2008; Lotte and Guan, 2011) allow to visualize patterns of brain activity that discriminate the location of attention shifts. We found that spatial patterns associated to the most discriminant filters reflected the alpha-band power modulation as previously shown with MEG (Yamagishi et al., 2003, 2005; Wyart and Tallon-Baudry, 2008; Bahramisharif et al., 2010; Roijendijk et al., 2013) and EEG (Worden et al., 2000; Sauseng et al., 2005; Thut et al., 2006; Rihs et al., 2007, 2009; Cosmelli et al., 2011). Moreover, we clearly observed these modulations in single-trial ambiguous epochs filtered by the most discriminant filters (**Figure 4**, CSP-L1 and CSP-R1) learned from predictive epochs. The spatial patterns of these filters mapped onto each subject’s scalp suggest that EEG recordings in predictive and ambiguous trials share similar topography (**Figure 3**). In addition, the CSP filters learned in the predictive condition showed similar discrimination performance for the ambiguous condition. This interesting result suggests that the distribution of features did not depends on the coherence of the random-dot motion. Therefore, it raises a fundamental question about the brain activity patterns reflecting covert attention shifts in complex visual environments containing either predictive or ambiguous spatial information. Could these two types of environments induce visuospatial attention processes that actually share common sources in the posterior brain regions? To address this issue more precisely, the spatial distribution of the CSP patterns could be analyzed using a source localization approach (Gramfort et al., 2014).

### Decoding Covert Shifts of Attention

The classification accuracies reported in our paper are comparable with the state of the art in visuospatial attention studies aiming at decoding features at two opposite attended locations (van Gerven and Jensen, 2009; Treder et al., 2011; Roijendijk et al., 2013; Tonin et al., 2013). In this paper, the decoding performance was quite variable across subjects, with classification accuracies ranging between 50% (chance level) and 80% (**Figure 5**). Several studies of covert visuospatial attention (van Gerven and Jensen, 2009; Bahramisharif et al., 2010; Roijendijk et al., 2013) distinguish “good” and “bad” performers based on the decoding performances. Two main hypotheses account for the high variability in our decoding results. First, the random-dot motion coherence settings may have influenced the subjects’ capacity to anticipate target location. Indeed, the high level of ambiguity in the cues used in this study is markedly different from classical decoding studies using totally unambiguous arrow cues (van Gerven and Jensen, 2009; Treder et al., 2011; Tonin et al., 2013). It is likely that bad performers have difficulty in switching attention when visual cues are not reliable enough. A complementary hypothesis for the decoding variability concerns the level of target contrast. The difficulty in discriminating between the two target orientations may have a direct influence on the strength of the subsequent shift of attention (Dosher and Lu, 2000). In support of this hypothesis, the classification accuracy showed a negative trend with target contrast suggesting that subjects were more prone to shift attention when contrast was low (Roijendijk et al., 2013). Good performers would therefore correspond to subjects that reached low level of target contrast in the calibration procedure. Regardless of the variability between subjects, classification accuracies were quite similar for the decoding of predictive and ambiguous features in each subject. This result suggests that, in the present task, modulations of cerebral activity are the only reliable source of information to discriminate the location of spatial attention. Indeed, with ambiguous random-dot motions, this information cannot be predicted from the cue itself.

These results have direct consequences for the development of BCI based on visuospatial attention. In particular, they show that features extracted from trials in which the spatial cue is highly predictive of the target location can be used to discriminate the location of attention in trials with ambiguous cues. Such observations indicate that in an online protocol, the spatial location of a visual target in ambiguous trials could be selected directly from the decoded location of attention. This approach is expected to have two main outcomes. First, the spatial ER in ambiguous trials will be decreased. Second, the subject’s behavioral performance measured by the RT and ER will improve. Preliminary results in an online protocol fit with these assumptions (Trachel et al., 2013).

## Conflict of Interest Statement

The authors declare that the research was conducted in the absence of any commercial or financial relationships that could be construed as a potential conflict of interest.
